# Fiber Tractography-Guided MR-Guided Focused Ultrasound Pallidothalamic Tractotomy for Parkinson's Disease: A Case Report

**DOI:** 10.7759/cureus.110159

**Published:** 2026-06-03

**Authors:** Hiroki Hori, Tomokatsu Hori, Takaomi Taira

**Affiliations:** 1 Radiology, FJK Tokyo Hospital, Tokyo, JPN; 2 Neurosurgery, Tokyo Neurological Center Hospital, Tokyo, JPN; 3 Neurosurgery, FJK Tokyo Hospital, Tokyo, JPN

**Keywords:** diffusion tensor image, focused ultrasound (fus), pallidothalamic tract, parkinson' s disease, three-dimensional magnetic resonance image

## Abstract

Pallidothalamic tractotomy (PTT) ablation using MR-guided focused ultrasound (MRgFUS) is a potential treatment option for medically refractory Parkinson’s disease (PD). Because the PTT is surrounded by critical neural structures, precise stereotactic targeting is essential to minimize neurological adverse events. Diffusion tensor imaging (DTI) fiber tractography may improve targeting accuracy through direct visualization of the PTT. We report a single case of staged bilateral PTT ablation using MRgFUS in a 45-year-old woman with advanced PD. During the first procedure, DTI fiber tractography was used to visualize the PTT and determine the stereotactic target. During the second procedure, contralateral targeting was performed using mirrored anatomical coordinates derived from the initial tractography findings in order to maintain anatomical consistency. Motor symptoms improved substantially following both procedures. The Movement Disorder Society-Unified Parkinson’s Disease Rating Scale (MDS-UPDRS) Part III score improved from 24 to 11 after the first procedure and from 52 to 11 after the second procedure. No neurological adverse events were observed throughout the follow-up period. Although limited by the single-case design, this report suggests that DTI fiber tractography-guided targeting may improve the visualization and identification of the PTT during MRgFUS.

## Introduction

Recently, pallidothalamic tractotomy (PTT) has been reported as a neurosurgical treatment of choice with radiofrequency or focused MR-guided focused ultrasound (MRgFUS) method [1-4]. The concept of PTT is to interrupt the fibers of both the ansa lenticularis and the lenticular fasciculus from the globus pallidus internus (GPi) [5-7].

Because the PTT is surrounded by critical neural structures, including the subthalamic nucleus (STN), zona incerta, red nucleus, and thalamic nuclei, precise stereotactic targeting is essential during lesioning procedures. Inaccurate targeting may increase the risk of neurological adverse events, including speech disturbances, gait impairment, and motor complications resulting from unintended injury to adjacent structures [4,8,9]. The pallidothalamic tract cannot be directly visualized using conventional structural MRI alone, and stereotactic targeting has traditionally relied on indirect atlas-based anatomical coordinates. Accurate identification of the tract remains technically challenging using conventional stereotactic anatomical landmarks alone [4,10].

In contrast, diffusion tensor imaging (DTI)-based tractography may allow patient-specific visualization of the fiber tract trajectory and surrounding white matter anatomy. Recent advances in DTI fiber tractography may improve stereotactic target visualization and facilitate lesioning procedures involving the pallidothalamic tract [11-14].

Although tractography-assisted targeting has been explored in functional neurosurgery and deep brain stimulation, reports describing tractography-guided MRgFUS-PTT remain limited. In particular, the potential utility of tractography for individualized target localization during staged bilateral procedures has not been well described.

We hypothesized that tractography-guided targeting may facilitate anatomical identification of the PTT and assist stereotactic target localization during MRgFUS. Here, we report a case of staged bilateral PTT using tractography-guided MRgFUS in a patient with Parkinson’s disease (PD).

## Case presentation

Patient history

A 45-year-old woman diagnosed with PD in 2016 presented with progressive bradykinesia, rigidity, motor fluctuations, dyskinesia, postural instability, and frequent falls despite pharmacological therapy. Medications at presentation included levodopa/benserazide (Madopar; 0.25 g per dose, four times daily) and amantadine (0.1 g per dose, three times daily). The duration of medication efficacy had progressively shortened to approximately three hours during the year preceding referral. She particularly complained of marked bradykinesia involving the left lower extremity and prominent resting tremor. Because symptom control had become insufficient with medical therapy alone, the patient was referred for MRgFUS-based functional neurosurgical treatment. Preoperative evaluation demonstrated a skull density ratio of 0.46, indicating favorable suitability for MRgFUS treatment.

DTI acquisition and tractography reconstruction

DTI was performed using a 3.0-T MRI system (Ingenia Elition; Philips Healthcare, Best, The Netherlands) with single-shot two-dimensional echo-planar imaging. Imaging parameters were as follows: repetition time, 3500 ms; echo time, 84 ms; b-value, 1000 s/mm²; 33 diffusion encoding directions; slice thickness, 2.5 mm; flip angle, 90°; matrix size, 144 × 144; and field of view, 230 mm.

Fiber tractography reconstruction was performed using DSI Studio (Frank Yeh, Pittsburgh, PA, USA), implementing the generalized q-sampling imaging (GQI) algorithm after anatomical alignment with the International Consortium for Brain Mapping (ICBM) standard brain template. A three-dimensional atlas-based flexible region-of-interest (ROI) approach was employed to visualize the PTT, including fibers passing through the globus pallidus internus, globus pallidus externus, and thalamic regions toward the ipsilateral supplementary motor cortex. Fibers traversing the subthalamic nucleus and red nucleus were excluded from reconstruction.

Fiber tracking parameters included a minimum tract length of 30 mm and a maximum tract length of 200 mm, with tractography terminated after the generation of 1,000,000 tracts.

First procedure: right PTT ablation

Preoperative clinical evaluation was performed in January 2023, under the on-medication condition. The Movement Disorder Society-Unified Parkinson’s Disease Rating Scale (MDS-UPDRS) Part III score was 24 [15].

In January 2023, the patient underwent MRgFUS ablation targeting the right PTT. DTI fiber tractography was generated preoperatively by a radiologic expert (H.H.) to visualize the PTT (Figure 1).

**Figure 1:**
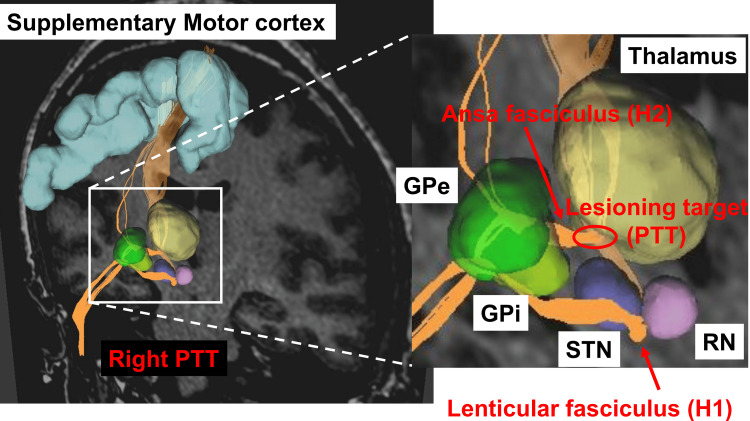
Tractography-based visualization of the pallidothalamic tract. Diffusion-based fiber tractography demonstrates the course of the right pallidothalamic tract. The pallidothalamic tractotomy (PTT) originates from the globus pallidus internus (GPi) and courses medially and dorsally toward the thalamus, circumventing the subthalamic nucleus (STN). Within the fields of Forel, pallidothalamic fibers are organized into the lenticular fasciculus (H1) and ansa lenticularis (H2), which converge before entering the motor thalamus. The therapeutic target was defined at the convergence of the H1 and H2 fields, where the tract is densely compacted. The image was generated using DSI Studio (freeware; Frank Yeh, Pittsburgh, PA, USA). Figure composition was performed using Microsoft PowerPoint (Microsoft Corporation, Redmond, Washington) without the use of generative artificial intelligence.

Moreover, tractography-derived projection images fused with three-dimensional T1-weighted MRI were used to measure the stereotactic target location relative to the posterior commissure (PC) (Figure 2).

**Figure 2:**
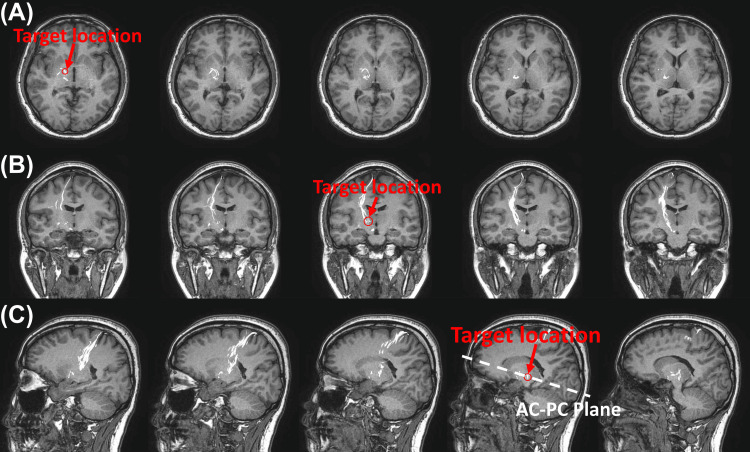
Two-dimensional projection of tractography onto three-dimensional T1-weighted MRI for target measurement (right PTT). (A) Axial, (B) coronal, and (C) sagittal views. DTI fiber tractography of the pallidothalamic tract was superimposed onto the patient’s three-dimensional T1-weighted MR images after anatomical alignment with the International Consortium for Brain Mapping (ICBM) standard brain template. The fused images were used to determine stereotactic target coordinates relative to the posterior commissure (PC). The target corresponded to the convergence zone of the H1 and H2 fields identified on tractography and was located 9.0 mm anterior to the PC, 8.0 mm lateral to the AC-PC midline on the right side, and at the level of the AC-PC plane.

Based on the tractography findings, the stereotactic target location was determined by an experienced stereotactic neurosurgeon using established anatomical landmarks.

The target was defined at 9.0 mm anterior to the PC, 8.0 mm lateral to the midline of the anterior commissure-posterior commissure (AC-PC) line on the right side, and at the level of the AC-PC plane. This location corresponded to the convergence zone of the H1 and H2 fields identified on tractography.

Thermal ablation was performed under MRI guidance using standard MRgFUS procedures. A total of six sonications were delivered, with a maximum energy of 24,128 J and a peak temperature of 56°C. The MRgFUS-induced lesion measured 8.5 × 8.6 × 10.4 mm in the anterior-posterior, right-left, and superior-inferior dimensions, respectively (Figure 3).

**Figure 3:**
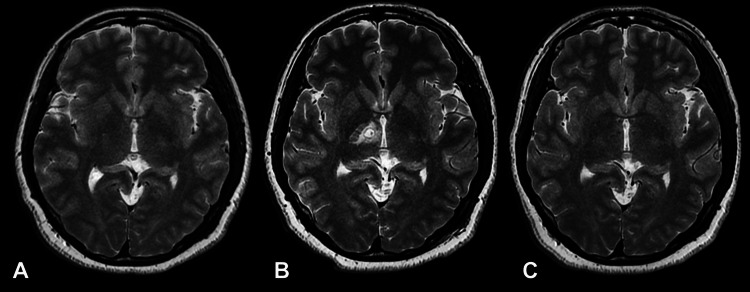
MR-guided focused ultrasound ablation of the right pallidothalamic tract (tractography-guided targeting). Axial T2-weighted MR images obtained before treatment (A), one day after treatment (B), and three months after treatment (C). The lesion is observed at the predefined target corresponding to the pallidothalamic tract identified using diffusion-based fiber tractography. The lesion appears as a focal hyperintense area on T2-weighted imaging and remains well circumscribed without evidence of extension into adjacent structures.

Postoperative evaluation performed in January 2023, while on medication, demonstrated improvement in motor function, with the MDS-UPDRS Part III score decreasing to 11. Follow-up evaluation in April 2023, under the off-medication condition, demonstrated an MDS-UPDRS Part III score of 40.

Second procedure: left PTT ablation

Because residual contralateral symptoms persisted, staged treatment of the left PTT was considered. Preoperative evaluation performed in February 2024, under the off-medication condition, demonstrated an MDS-UPDRS Part III score of 52.

In February 2024 (13 months after the first procedure), MRgFUS ablation of the left PTT was performed. DTI fiber tractography was not repeated during the second procedure. Instead, the target location was determined by an experienced stereotactic neurosurgeon (T.H., T.T.) using mirrored anatomical coordinates corresponding to the previously identified right PTT location.

The target was defined at 11.0 mm anterior to the PC, 9.0 mm lateral to the midline of the AC-PC line, and 1.5 mm superior to the AC-PC plane. Four sonications were administered during the left-sided procedure, with a maximum delivered energy of 25,113 J and a peak temperature of 57°C. The resulting lesion measured 7.8 × 8.2 × 7.4 mm in the anterior-posterior, right-left, and superior-inferior dimensions, respectively (Figure 4).

**Figure 4:**
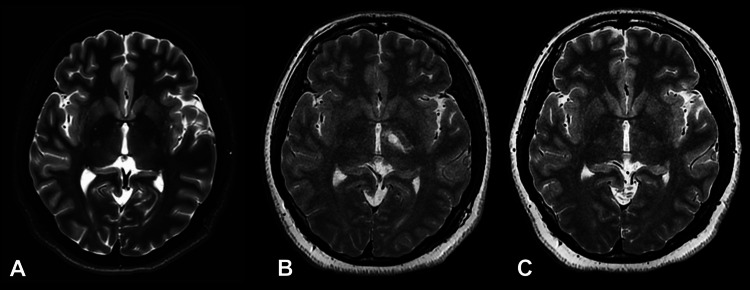
MR-guided focused ultrasound ablation of the left pallidothalamic tract (mirror-based targeting). Axial T2-weighted MR images obtained before treatment (13 months after right-sided ablation) (A), one day after treatment (13 months after right-sided ablation) (B), and three months after treatment (20 months after right-sided ablation) (C). The target location was determined using mirrored anatomical coordinates based on the previously identified right pallidothalamic tract. The lesion is observed at the corresponding location and appears as a focal, well-circumscribed hyperintense area on T2-weighted imaging without evidence of extension into adjacent structures.

Postoperative evaluation performed in February 2024, under the off-medication condition, demonstrated marked motor improvement, with the MDS-UPDRS Part III score decreasing to 11. Following the left-sided procedure, the MDS-UPDRS Part III score improved from 52 to 11 under the off-medication condition, representing a 78.8% reduction in motor symptom severity.

Follow-up after bilateral treatment

Long-term follow-up evaluations were performed approximately one year after the second procedure. In February 2025, evaluation under the off-medication condition demonstrated an MDS-UPDRS Part III score of 34. In February 2025, evaluation under the on-medication condition demonstrated further improvement, with a score of 5 (Figure 5).

**Figure 5:**
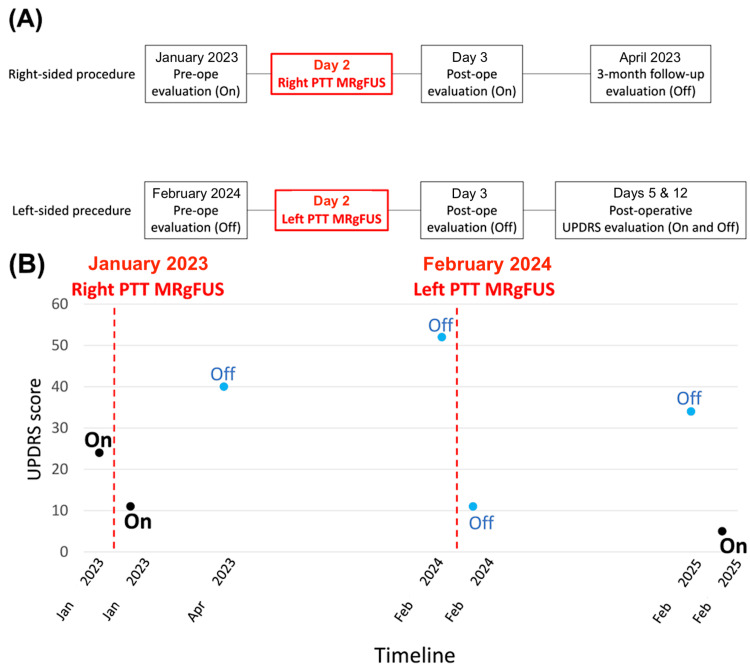
Clinical timeline and longitudinal changes in Movement Disorder Society-Unified Parkinson’s Disease Rating Scale (MDS-UPDRS) Part III scores following staged bilateral pallidothalamic tract ablation using MR-guided focused ultrasound. (A) Clinical timeline showing staged right- and left-sided PTT procedures, serial MDS-UPDRS Part III evaluations, and long-term follow-up assessments. (B) Longitudinal changes in MDS-UPDRS Part III scores following staged bilateral MRgFUS PTT. Vertical dashed lines indicate the timing of each procedure. Medication status (on or off) is shown adjacent to each data point. ON-medication assessments are shown in black and OFF-medication assessments in blue. Sustained motor improvement was observed after both procedures. The figure was created using Microsoft PowerPoint and Excel (Microsoft Corporation, Redmond, Washington) without the use of generative artificial intelligence. Copyright © 2014-2019 International Parkinson and Movement Disorder Society (MDS). All Rights Reserved. Reproduced with permission of the copyright owner. Further reproduction is prohibited without permission.

Throughout the follow-up period, the patient did not develop neurological complications, including speech disturbance, gait impairment, cognitive decline, or other treatment-related adverse events. Longitudinal medication adjustments after treatment were unavailable because follow-up pharmacological management was performed outside our institution.

CARE guidelines

This case report was prepared in accordance with the CARE reporting guidelines.

## Discussion

This case demonstrates the potential utility of DTI fiber tractography for facilitating visualization and stereotactic targeting of the pallidothalamic tract during MRgFUS. Because the pallidothalamic region is anatomically complex and surrounded by eloquent neural structures, precise targeting is essential to achieve safe and accurate ablation [1-4,10,12].

In the present case, the therapeutic target was defined at the convergence zone of the H1 and H2 fields, where pallidothalamic fibers become densely compacted. Targeting this anatomical region may allow for efficient modulation of pathological pallidal output while minimizing lesion volume and reducing involvement of adjacent neural structures.

Traditionally, stereotactic targeting of the pallidothalamic region has relied primarily on indirect anatomical coordinates referenced to the AC-PC line [1-4]. However, interindividual anatomical variability may limit the precision of coordinate-based targeting alone. Recent advances in DTI fiber tractography now enable visualization of white matter pathways in vivo and provide additional anatomical information regarding the spatial relationship between the PTT and surrounding structures [11,13,16-19]. This workflow enabled individualized target planning based on direct visualization of the tract trajectory rather than reliance solely on atlas-based anatomical estimation.

DTI fiber tractography may also have broader applicability in other stereotactic functional neurosurgical procedures, including radiofrequency lesioning and deep brain stimulation, where improved visualization of patient-specific white matter anatomy may assist target identification and reduce unintended involvement of adjacent eloquent structures [7,11-14].

Despite its potential utility, diffusion tensor tractography has inherent technical limitations, including susceptibility to reconstruction variability, operator dependency, and false-positive or false-negative fiber representation. Complex fiber crossing and partial volume effects may influence tract visualization accuracy, particularly in anatomically compact regions such as the PTT. Therefore, tractography findings should be interpreted in conjunction with conventional anatomical landmarks and stereotactic expertise [18,19].

Importantly, no neurological adverse events were observed despite staged bilateral intervention. Although the present report cannot establish causality, precise tractography-guided targeting may have reduced collateral injury to adjacent eloquent structures during stereotactic lesioning procedures. The lesions remained focal and well-circumscribed on follow-up MRI, without radiological evidence of extension into surrounding tissue. In addition, substantial motor improvement was observed following both procedures, with sustained clinical benefit at one-year follow-up after bilateral treatment.

The second procedure was performed using mirrored anatomical coordinates derived from the previously identified right-sided PTT without repeating tractography. Although this approach assumes relative anatomical symmetry of the PTT, no neurological complications were observed following the second procedure. Nevertheless, potential interhemispheric anatomical variability cannot be excluded and warrants further validation in larger studies.

Several limitations should be acknowledged. First, this report describes a single patient and therefore does not permit conclusions regarding efficacy or safety. Second, clinical evaluations were performed under mixed medication conditions because follow-up assessments were conducted at different time points and institutions. Third, interpretation of longitudinal MDS-UPDRS Part III changes should be performed with caution because evaluations were obtained under mixed on- and off-medication conditions, depending on the patient’s clinical circumstances and the international follow-up setting. Detailed longitudinal data on antiparkinsonian medication adjustments were unavailable during follow-up, which may have influenced the interpretation of long-term clinical outcomes. Finally, quantitative radiological validation, including lesion overlap analysis and postoperative tract integrity assessment, was not performed in this single-case report. Future studies with standardized imaging analysis may help clarify the anatomical accuracy of tractography-guided targeting.

Despite these limitations, this case highlights the potential value of DTI fiber tractography-guided targeting during MRgFUS PTT ablation. Direct visualization of the PTT may improve stereotactic accuracy and facilitate stereotactic lesioning procedures in anatomically complex regions. Further studies involving larger patient cohorts are needed to clarify the clinical utility, reproducibility, and long-term safety of tractography-guided PTT ablation in patients with PD.

## Conclusions

This case suggests that DTI fiber tractography-guided targeting during MRgFUS PTT is technically feasible and may facilitate individualized stereotactic targeting in anatomically complex regions. Staged bilateral PTT ablation was associated with substantial motor improvement without major neurological complications. Further studies are required to evaluate the reproducibility, safety, and long-term clinical utility of tractography-guided PTT ablation in patients with PD.
